# m-Power Heart Project - a nurse care coordinator led, mHealth enabled intervention to improve the management of hypertension in India: study protocol for a cluster randomized trial

**DOI:** 10.1186/s13063-018-2813-2

**Published:** 2018-08-07

**Authors:** Nikhil Srinivasapura Venkateshmurthy, Vamadevan S Ajay, Sailesh Mohan, Devraj Jindal, Shuchi Anand, Dimple Kondal, Nikhil Tandon, Malipeddi Bhaskara Rao, Dorairaj Prabhakaran

**Affiliations:** 1 Public Health Foundation of India, Plot 47, Sector 44, Gurgaon, India; 2 Center for Chronic Disease Control, C-1/52, 2nd Floor, Safdarjung Development Area, New Delhi, India; 3 Division of Nephrology, Stanford University School of Medicine, Palo Alto, CA USA; 4 Department of Endocrinology and Metabolism, All India Institute of Medical Sciences, New Delhi, India; 5 KIMS Hospital, Visakhapatnam, India

**Keywords:** Hypertension, Chronic kidney disease (CKD), Nurse care coordinator (NCC), Electronic decision support system (EDSS), Task-sharing, Public health system, India

## Abstract

**Background:**

The proportion of patients with controlled hypertension (< 140/90 mmHg) is very low in India. Thus, there is a need to improve blood pressure management among patients with uncontrolled hypertension through innovative strategies directed at health system strengthening.

**Methods:**

We designed an intervention consisting of two important components – an electronic decision support system (EDSS) used by a trained nurse care coordinator (NCC). Based on preliminary data, we hypothesized that this intervention will be able to reduce mean systolic blood pressure by 6.5 mmHg among those with uncontrolled blood pressure in the intervention arm compared to the standard treatment arm (paper-based hypertension treatment guidelines). The study will adopt a cluster randomized trial design with the community health center (CHC) as the unit of randomization. The trial will be conducted in Visakhapatnam district (southern India). A total of 1876 participants aged ≥30 years with high blood pressure – systolic blood pressure (SBP) ≥ 160 mmHg or diastolic blood pressure (DBP) ≥ 90 mmHg will be enrolled from 12 CHCs. The intervention consists of trained NCCs equipped with an evidence-based hypertension treatment algorithm in the form of the EDSS with regular SMSs to patients with hypertension to promote hypertension treatment and blood pressure control for 12 months. The primary outcome will be difference in the mean change of SBP, from baseline to 12 months, between the intervention and the standard treatment arm. The secondary outcomes are the difference in mean change of DBP; difference in the proportion of patients with controlled blood pressure (< 140/90 mmHg); difference in mean change of fasting blood sugar, HbA1C, eGFR, and albumin to creatinine ratio; difference in the proportion of patients visiting the CHC regularly (number of actual visits to the CHC/number of visits suggested by the EDSS > 80%); difference in proportion of patients compliant to anti-hypertensive medication/s; cost-effectiveness of intervention versus enhanced care. All the outcomes will be assessed at 12 months.

**Discussion:**

The study is expected to provide evidence on the effectiveness of NCC-led, EDSS-based hypertension management in India and can likely offer an exemplar for improving cardiovascular disease (CVD) management in India within the resource-constrained public healthcare system.

**Trial registration:**

ClinicalTrials.gov, ID: NCT03164317). Registered retrospectively on 23 May 2017 (first patient enrolled on 6 April 2017) because the authors did not receive a response to their original registration submission (5 January 2017) to the Clinical Trial Registry – India (CTRI).

**Electronic supplementary material:**

The online version of this article (10.1186/s13063-018-2813-2) contains supplementary material, which is available to authorized users.

## Background

Non-communicable diseases (NCDs) are the number-one cause of death in India [1]. Cardiovascular diseases (CVDs) cause the majority of deaths among NCDs. High blood pressure or hypertension is one of the important risk factors for CVD. The prevalence of hypertension is rising steadily – both in urban as well as rural India. Estimates suggest that out of 10 patients with hypertension only one has their blood pressure meeting recommended targets [2]. Thus, there exists a huge need and potential to improve management among those with uncontrolled blood pressure.

The public healthcare system in India is overburdened, and has yet to reorient fully to address NCDs. The busy out-patient settings of the public healthcare facilities offer little time for the physicians to provide optimal treatment and care for patients with hypertension, who need a steady medication supply as well as intensive teaching on diet and medication compliance. Task-shifting/sharing, a process wherein certain tasks can be delegated to less qualified but appropriately trained paramedical workers, like nurses [3], offers a potential solution to improve the sub-optimal management of hypertension. mHealth, i.e., the use of mobile and wireless technologies to support the achievement of health objective [4] technology has shown promising results with improvement in medication adherence for NCD treatment [5], physical activity and promotion of healthy diets among patients with NCDs [6]. Thus, mHealth can be leveraged to equip the nurses with the tools to assist the physicians in managing blood pressure.

Our pilot study, with a pre-post design, implemented in northern India, has previously shown that this model can achieve substantial reductions in blood pressure and blood sugar [7]. The intervention consisted of task-sharing with an electronic decision support system (EDSS) to aid physicians in the management of blood pressure of patients with uncontrolled hypertension. The reductions in systolic blood pressure (SBP) and diastolic blood pressure (DBP) at the end of 18 months were 14.6 mmHg and 7.6 mmHg, respectively. However, this pilot study lacked a comparator group. In order to rigorously test the feasibility and effectiveness of this intervention, i.e., a trained NCC who uses an EDSS to guide blood pressure treatment in a public healthcare setting, we plan to conduct a cluster randomized control trial involving patients visiting the community health centers (CHCs) of the southern Indian state of Andhra Pradesh.

## Methods

### Trial design

**Fig. 1:**
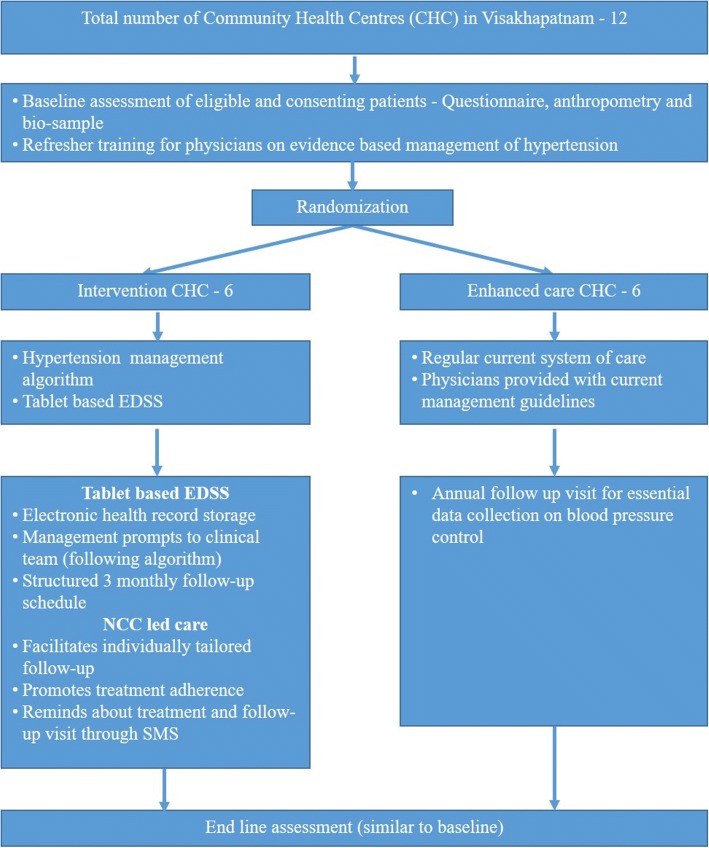
Schema of the m-Power Heart Project. Footnote: EDSS – electronic decision support system

The study will be a parallel-group, cluster randomized trial to test the effectiveness of a trained NCC-led intervention by comparing with usual care. The trial design schema is depicted in Fig. 1. The corresponding Standard Protocol Items: Recommendations for Interventional Trials (SPIRIT) 2013 Checklist [8] is provided as Additional file 1.

### Trial site selection

The study will be carried out in the CHCs of Visakhapatnam district which has a population of approximately 4,000,000. The district has three distinct geographical divisions – urban, rural and tribal. Nearly 46% of the total population live in the city of Visakhapatnam. A CHC is a secondary-level public healthcare facility providing referral services and caters to a population of 80,000 to 120,000. As per the Indian Public Health Standards (IPHS) [9], a CHC has to provide specialty services (general medicine, general surgery, pediatrics, obstetrics and gynecology, and anesthesia) and is headed by a block medical officer or a medical superintendent. The list of all the CHCs in Visakhapatnam district was obtained from the office of the District Coordinator of Hospital Services (DCHS), Visakhapatnam. A total of 13 CHCs are functioning in Visakhapatnam district. In one CHC, a comprehensive diabetes and hypertension prevention and management program, titled UDAY, is already being implemented. We obtained permission from the DCHS to implement the m-Power Heart Project in the remaining 12 CHCs.

### Eligibility criteria

All 12 CHCs will be eligible for participating in the study. The participant inclusion and exclusion criteria are listed in Table 1.

**Table 1:** Inclusion and exclusion criteria of study participants

Inclusion criteria	1. Age ≥ 30 years 2. On treatment for hypertension or opportunistically screened and diagnosed with hypertension, with a blood pressure of ≥160/90 mmHg 3. With or without co-morbidities (diabetes mellitus, left ventricular hypertrophy, chronic kidney disease, heart failure, coronary artery disease, and peripheral vascular disease)
Exclusion criteria	1. Pregnant women 2. Unwilling/unable to provide written informed consent for the study 3. Diagnosed with a malignancy or life-threatening condition 4. Currently enrolled in other trials 5. Plans to move residence in the year ahead

### Intervention

The intervention has two important components. The first is the EDSS which has been developed as an android application installed in a tablet. The EDSS uses an algorithm developed by national and international experts, based on national and international guidelines [10–13] for the management of hypertension. The EDSS considers a patient’s current age, blood pressure level, any co-morbid conditions and current medication (type, dose, and frequency) to suggest to the physician the best course of treatment. For example, consider that a male patient aged 58 years, taking amlodipine (5 mg once daily), has a SBP of 160 mmHg and a diastolic blood pressure (DBP) of 90 mmHg and no other co-morbidities. The EDSS recommends to the physician to add enalapril 5 mg or losartan 50 mg once daily. The EDSS also screens patients for chronic kidney disease (CKD) by calculating the estimated glomerular filtration rate (eGFR) based on current serum creatinine value [14] and alerting the physician to refer high-risk patients (e.g., eGFR < 30 ml/min/1.73 m^2^) to a higher level of care (i.e., district hospital in Visakhapatnam) where the services of a nephrologist are available for the management of CKD. Apart from the clinical recommendation, the EDSS also recommends lifestyle changes (reduction of salt, increase in consumption of fruits and vegetables, quitting tobacco and alcohol). The EDSS will facilitate patient data management by storing the information electronically (which is currently not available in the CHCs) and also provide a structured follow-up plan for each patient.

The second important component of the intervention is the NCC-led care. The task of management of hypertension is shared with the physician by the NCC. The NCC will measure blood pressure, provide counseling on lifestyle changes, facilitate treatment and follow-up and promote adherence to the prescribed treatment and follow-up schedule. The NCCs will receive training in the diagnosis and management of hypertension and its co-morbidities, and in the use of EDSS. The NCC will be supported by an automated short messaging service (SMS) that will regularly send messages in the local language to patients enrolled in the study. The SMS will convey information on various aspects of hypertension and its management – risk factors, importance of lifestyle changes, regular intake of medication, reminders on follow-up visits, etc. A bank of approximately 50 messages will be developed in English in consultation with study investigators and experts in the field of hypertension management and translated into the local language for transmission to the patients.

### Usual care

In the standard treatment arm CHC, the regular and routine care will continue. The physicians will be provided with paper-based current evidence-based management guidelines. The physicians in both the arms will receive refresher training on hypertension management prior to the randomization of CHCs.

### Outcomes

#### Primary outcome

- Difference in the mean change of SBP, from baseline to 12 months, between the intervention and enhanced care arms

#### Secondary outcome(s)

- i Difference in the mean change from baseline to 12 months between the intervention and enhanced care arms in:
  - ◦ DBP
  - ◦ Fasting blood sugar
  - ◦ Glycated hemoglobin (HbA1C)
  - ◦ Blood urea and serum creatinine
  - ◦ eGFR and albumin to creatinine ratio
- ii Difference in the proportion of patients from baseline to 12 months between the intervention and enhanced care arms in:
  - ◦ Controlled blood pressure (< 140/90 mmHg)
  - ◦ Albuminuria (urine albumin to creatinine ratio > 30 mg/g)
  - ◦ Visiting the CHC regularly (number of actual visits to the CHC/number of visits suggested by the EDSS > 80%)
  - ◦ Compliance to anti-hypertensive medication/s
- iii Cost-effectiveness of the intervention compared to standard treatment

All outcomes will be assessed at the end of the intervention, i.e., 12 months.

### Participant timeline

**Fig. 2:**
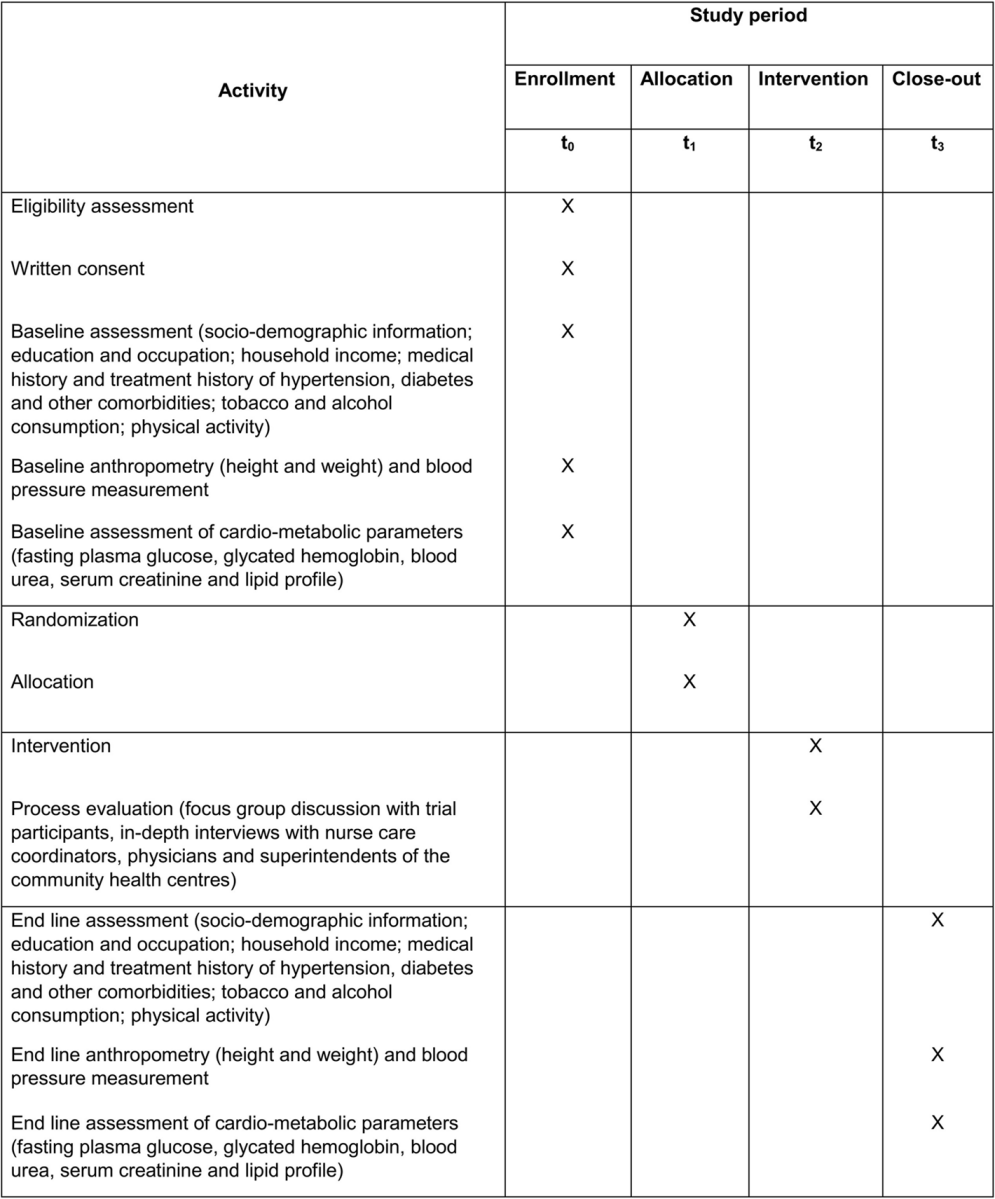
Schedule of enrollment, assessments, and intervention

The participant timeline is provided in Fig. 2.

### Sample size

The sample size has been calculated based on the following – significance at 5%, power of 80%, mean difference in SBP of 6.5 mmHg between the intervention and standard treatment arms, standard deviation (SD) of SBP of 17, intracluster coefficient of 0.03 (estimated from the baseline study of a comprehensive diabetes and hypertension prevention and management program entitled UDAY in Visakhapatnam) and attrition rate of 20%. The sample size will be 936 participants from six CHCs in each arm, which totals to 1872 from 12 CHCs.

### Randomization and blinding

Randomization will be at the level of the CHC in a ratio of 1:1 and will be carried out by a statistician based at the Public Health Foundation of India (PHFI) in Gurgaon, India. The randomization will be done only after the baseline assessment of the eligible participants and the completion of refresher training of all the physicians in the CHC in order to maintain the balance between the two arms prior to the intervention roll out. CHCs will be assigned to either the intervention or the standard treatment arm by the statistician. Participants will be enrolled by the NCC posted at CHCs. The blinding of either the study participants or the NCC is not possible. The imbalance for the baseline covariates leads to poor internal validity and reduced study power/precision of estimates. The design is based on adjustment of baseline covariates to avoid chance imbalance. Covariate-constrained randomization [15, 16] is the method which is used to balance for many covariates between the intervention and standard treatment arms. This method limits the risk of selection bias. In this study we propose to balance both arms by baseline cluster-level covariates; i.e., the proportion of women, education level, proportion of newly detected cases of hypertension and proportion of CKD cases. The STATA program (cvcrand) will be used to implement covariate-constrained randomization.

### Recruitment

Patients with known hypertension attending the out-patient clinics will be approached and the study objectives will be explained to them. If they meet the eligibility criteria, they will be asked to provide their consent to participate. If they provide consent, baseline questionnaire administration, anthropometry, and bio-sample collection will be carried out. Apart from patients with known hypertension, all other patients aged ≥ 30 years visiting the out-patient clinic for complaints other than hypertension will be screened via blood pressure measurement and recruited as mentioned above. Recruitment of the participants will be prior to randomization. To achieve the target recruitment numbers, co-operation of the medical superintendent, physicians, and the administrative staff of CHC will be sought. They will be oriented to the procedures of recruitment and the trial. Information about the trial and the location of NCCs will be displayed at the registration counter, out-patient chambers and other prominent areas of the CHCs to help patients navigate and find the NCC chamber.

### Data collection and management

Baseline and endline assessment of study participants will be conducted. Data on key sociodemographic variables (age, sex, education, employment, and household income), duration of, and treatment for, hypertension, and lifestyle (diet, physical activity, tobacco and alcohol) behaviors will be collected electronically. Blood and urine samples will be collected and analyzed for key cardio-metabolic parameters (fasting blood sugar, HbA1C, blood lipids, blood urea, serum creatinine, urine albumin to creatinine ratio). Blood pressure, height and weight will be measured. The NCCs will be trained to administer the questionnaire and carry out the measurements. A trained phlebotomist will be employed for blood sample collection. All samples collected will be analyzed in an accredited local laboratory based on standard laboratory procedures.

The data collected will be stored in the electronic server placed at PHFI in Gurgaon, India. The server is maintained by a dedicated team with regular backup procedures in place. The access to the information in the server will be restricted and made available only to the researchers associated with the m-Power Heart Project. Personal identifiers will be removed prior to analysis. Instead, participant identification numbers will be used which is not meaningful to casual observers without access to the original study logs (accessible only to the permitted research team). All data files will be maintained under password protection at all times. The study records will be available to the Institutional Ethics Committee (IEC).

During the baseline and endline assessments, data will be monitored daily by the project manager. At the end of each day, a data dump will be taken from the server and checked for missing values, errors, and inconsistencies. If found, the information will be passed on to the concerned NCC and they will be asked to correct the information prior to carrying out the next interview.

Ischemic heart disease, stroke, end-stage renal disease (ESRD) or death will be treated as serious adverse events and recorded by the NCC during each visit to the CHC. Information on the death of the participants will be collected from the contacts (relatives/friends listed by the participant during the baseline assessment).

### Statistical analysis

The continuous outcome measured at baseline and end of follow-up (SBP) will be analyzed using individual-level data with a linear mixed-effects model where the dependent variable will be the difference in outcome (after-before) and the main explanatory variable will be the treatment group. The model will include a random effect of the CHC to account for the clustered design. The model will be adjusted by baseline outcome values in two ways: Firstly, the subject’s “regression to mean effect” will be controlled by including distance to the CHC mean of the subject’s baseline outcome value. Secondly, we will also include the CHC’s baseline outcome mean to adjust for the baseline differences between CHCs. The outcome measurements (baseline and final) will be suitably transformed to achieve normality and to deal with outliers depending on their distribution, and they will be centered on the mean of the baseline measure in each trial arm separately. Such a model will effectively test if the mean changes in the outcome variable during the study period are different between the two study arms while considering the cluster design and the potential regression to the mean effect. Estimations of the parameters will be reported along with the 95% confidence intervals and *p* values for the null value.

The covariate-constrained randomization will be used to balance between the intervention and control clusters. If there is imbalance between the intervention and standard treatment arms, then the analysis will be adjusted for these variables. There is no statistical test for confounding to decide which variables should enter the model, so judgment will have to be made based on the actual imbalances between arms and the association of those variables with the outcomes. There will be full transparency and the final results will be presented for the models unadjusted and adjusted by the baseline variables that are considered important due to their imbalances between arms. In addition to that, all cluster-level covariates used in the constrained randomization procedure will be adjusted in the analysis.

The primary analyses will be conducted under the principle of intention-to-treat. All randomized patients will be analyzed in the groups to which they were originally allocated, regardless of whether they retained that specific group membership over the course of the trial or not. Patients who withdrew consent for use of their data will not be included in any analyses.

Dropouts and lost-to-follow-up are a concern in any trial. We will compare the baseline characteristics between those who drop out and those who stay in the trial. It is very difficult to decide the best way to deal with this issue a priori before we can verify the extent of the problem in the final dataset. In general, the approach for each outcome will be to perform and present both: complete case analysis and multiple imputation analysis and, if there are substantial differences in the results, we will discuss which one seems more reasonable given the characteristics of the missing data. We will also consider other subgroup analyses based on other variables such as co-morbid diabetes, co-morbid CKD and new versus prior diagnosis of hypertension. The findings will be reported according to the Consolidated Standards of Reporting Trial (CONSORT) guidelines [17].

### Process evaluation

The Consolidated Framework For Implementation Research (CFIR) [18] will be used to evaluate the process of implementation of the trial. The aim of the process evaluation is to understand the contextual factors under which the intervention was implemented, the reasons behind the success/failure of the intervention and the challenges faced by the NCCs in implementation of the intervention. The results of the process evaluation will contribute to better understanding of the process and aid in potential scale-up of the intervention to other similar settings.

The process evaluation will employ qualitative methods. Focus group discussions (FGDs) with the patients visiting the CHCs, key informant interviews with the physicians and superintendents of the CHCs and in-depth interviews with NCCs will be conducted. All interviews and discussion will be audio-recorded after obtaining the requisite permission from the respective participants. The recordings will be transcribed and translated as soon as possible. Descriptive content analysis will be carried out. The decision will be taken on coding rules and theme generation after consensus among the investigators. Inductive as well as deductive codes will be generated. Similar codes will be clubbed together into themes. The themes will be described. The findings will be reported by using Consolidated Criteria for Reporting Qualitative Research [19].

### Publication and dissemination of the results

The results of the trial will be presented in relevant national and/or international conferences and published in a peer-reviewed journal. The results will also be disseminated through policy briefs and stakeholder meetings. Print, electronic, and social media will be utilized to share the results to non-specialist audiences.

## Discussion

To the best of our knowledge, the m-Power Heart Project is the first study to evaluate the effectiveness of an NCC-led, mHealth-enabled intervention on blood pressure control using a cluster randomized trial design. Three previous studies in primary care settings of India have demonstrated the effectiveness of the mHealth intervention [7, 20, 21]. In the study by Anchala et al. [20], the decision support system was managed by the physicians and there were no NCCs to support them. The SimCard trial [21] intervention followed a simple 2 + 2 model – smoking cessation and salt reduction plus anti-hypertensive medication (calcium channel blocker 2.5/5 mg per day) and aspirin (75 to 100 mg per day). In the m-Power intervention study [7], the SBP reduced by 12.9 mmHg (95% CI: − 13.2 to − 12.7) in 3 months and by 14.6 mmHg (95% CI: − 15.3 to − 13.8) in 18 months. In our study, we are testing the effectiveness of EDSS through trained NCCs to decrease SBP by 6.5 mmHg in the intervention arm compared to the control. NCC together with EDSS is likely to ease the burden of hypertension management on already over-worked physicians in the public healthcare system. Also, the intervention in the m-Power Heart Project is more comprehensive than the SimCard trial which includes hypertension management (based on more than one class of anti-hypertensive and considering patient co-morbidities); screening, management, and referral for CKD and SMS reminders.

Our study is timely and relevant. In June 2016, the guidelines for the screening of hypertension, together with other NCDs, was released by the Government of India. The National Program for Prevention and Control of Cancer, Diabetes, Cardiovascular Disease, and Stroke (NPCDCS) recommends the setting up of an “NCD Clinic” at the CHC [22]. Various activities, like diagnosis and management, health promotion, referral, data recording and reporting, are to be carried out by personnel appointed, which includes two nurses along with a medical officer. The m-Power application and the structured training of nurses will aid in better management of patients visiting the CHC for care and subsequent reduction of blood pressure resulting in improved health outcomes. We hope that the evidence generated through the trial will contribute to gaining acceptance and wider use of the EDSS and task-shifting in India’s public healthcare system.

### Trial status

Recruitment is on-going.

## Additional file

Additional file 1: Standard Protocol Items: Recommendations for Interventional Trials (SPIRIT) 2013 Checklist: recommended items to address in a clinical trial protocol and related documents*. (DOCX 49 kb)

## Abbreviations

CCDC: Center for Chronic Disease Control
CHC: Community health center
CKD: Chronic kidney disease
CVDs: Cardiovascular diseases
DCHS: District Coordinator of Hospital Services
EDSS: Electronic decision support system
eGFR: Estimated glomerular filtration rate
ICMR: Indian Council of Medical Research
IPHS: Indian Public Health Standards
NCC: Nurse care coordinator
NCDs: Non-communicable diseases
NPCDCS: National Program for Prevention and Control of Cancer, Diabetes, Cardiovascular Disease, and Stroke
PHFI: Public Health Foundation of India
SBP: Systolic blood pressure
SMS: Short messaging service

## References

[1] WHO (2015). Noncommunicable diseases progress monitor 2015.

[2] Anchala R, Kannuri NK, Pant H, Khan H, Franco OH, Di Angelantonio E, Prabhakaran D (2014). Hypertension in India: a systematic review and meta-analysis of prevalence, awareness, and control of hypertension. J Hypertens.

[3] WHO (2008). Task shifting: rational redistribution of tasks among health workforce teams: global recommendations and guidelines.

[4] WHO (2011). mHealth: new horizons for health through mobile technologies: second global survey on eHealth.

[5] Stephani V, Opoku D, Quentin W (2016). A systematic review of randomized controlled trials of mHealth interventions against non-communicable diseases in developing countries. BMC Public Health.

[6] Muller AM, Alley S, Schoeppe S, Vandelanotte C (2016). The effectiveness of e-& mHealth interventions to promote physical activity and healthy diets in developing countries: a systematic review. Int J Behav Nutr Phys Act.

[7] Ajay VS, Jindal D, Roy A, Venugopal V, Sharma R, Pawar A, Kinra S, Tandon N, Prabhakaran D. Development of a Smartphone-enabled hypertension and diabetes mellitus management package to facilitate evidence-based care delivery in primary healthcare facilities in India: the mPower Heart Project. J Am Heart Assoc. 2016;5(12) 10.1161/JAHA.116.004343. 10.1161/JAHA.116.004343 PMC5210443 28003248

[8] Chan AW, Tetzlaff JM, Gotzsche PC, Altman DG, Mann H, Berlin JA, Dickersin K, Hrobjartsson A, Schulz KF, Parulekar WR (2013). SPIRIT 2013 explanation and elaboration: guidance for protocols of clinical trials. BMJ.

[9] Pronovost PJ, Berenholtz SM, Goeschel CA, Needham DM, Sexton JB, Thompson DA, Lubomski LH, Marsteller JA, Makary MA, Hunt E (2006). Creating high reliability in health care organizations. Health Serv Res.

[10] Indian Guidelines on Hypertension (IGH) 3 (2013). Management of hypertension. J Assoc Phys Ind.

[11] WHO (2007). Guidelines for assessment and management of cardiovascular risk.

[12] Mancia G, Fagard R, Narkiewicz K, Redon J, Zanchetti A, Bohm M, Christiaens T, Cifkova R, De Backer G, Dominiczak A (2013). 2013 ESH/ESC guidelines for the management of arterial hypertension: the Task Force for the Management of Arterial Hypertension of the European Society of Hypertension (ESH) and of the European Society of Cardiology (ESC). Eur Heart J.

[13] James PA, Oparil S, Carter BL, Cushman WC, Dennison-Himmelfarb C, Handler J, Lackland DT, LeFevre ML, MacKenzie TD, Ogedegbe O (2014). 2014 evidence-based guideline for the management of high blood pressure in adults: report from the panel members appointed to the Eighth Joint National Committee (JNC 8). JAMA.

[14] Levey AS, Stevens LA, Schmid CH, Zhang YL, Castro AF, Feldman HI, Kusek JW, Eggers P, Van Lente F, Greene T (2009). A new equation to estimate glomerular filtration rate. Ann Intern Med.

[15] Dickinson LM, Beaty B, Fox C, Pace W, Dickinson WP, Emsermann C, Kempe A (2015). Pragmatic cluster randomized trials using covariate constrained randomization: a method for practice-based research networks (PBRNs). J Am Board Fam Med.

[16] Li F, Turner EL, Heagerty PJ, Murray DM, Vollmer WM, DeLong ER (2017). An evaluation of constrained randomization for the design and analysis of group-randomized trials with binary outcomes. Stat Med.

[17] Campbell MK, Piaggio G, Elbourne DR, Altman DG, Group C (2012). CONSORT 2010 Statement: extension to cluster randomised trials. BMJ.

[18] Damschroder LJ, Aron DC, Keith RE, Kirsh SR, Alexander JA, Lowery JC (2009). Fostering implementation of health services research findings into practice: a consolidated framework for advancing implementation science. Implement Sci.

[19] Tong A, Sainsbury P, Craig J (2007). Consolidated criteria for reporting qualitative research (COREQ): a 32-item checklist for interviews and focus groups. Int J Qual Health Care.

[20] Anchala R, Kaptoge S, Pant H, Di Angelantonio E, Franco OH, Prabhakaran D (2015). Evaluation of effectiveness and cost-effectiveness of a clinical decision support system in managing hypertension in resource constrained primary health care settings: results from a cluster randomized trial. J Am Heart Assoc.

[21] Tian M, Ajay VS, Dunzhu D, Hameed SS, Li X, Liu Z, Li C, Chen H, Cho K, Li R (2015). A cluster-randomized, controlled trial of a simplified multifaceted management program for individuals at high cardiovascular risk (SimCard trial) in rural Tibet, China, and Haryana, India. Circulation.

[22] Operational guidelines. Prevention, screening and control of common non-communicable diseases: Hypertension, diabetes and common cancers (Oral, Breast, Cervix). New Delhi: Ministry of Health and Family Welfare, Government of India; 2017.

